# Co-Occurrence of Plasmid-Mediated AmpC β-Lactamase Activity Among *Klebsiella pneumoniae* and *Escherichia Coli*

**DOI:** 10.2174/1874285801711010195

**Published:** 2017-09-26

**Authors:** Abdulaziz Zorgani, Hiyam Daw, Najib Sufya, Abdullah Bashein, Omar Elahmer, Chedly Chouchani

**Affiliations:** 1 Medical Microbiology and Immunology Department, Faculty of Medicine, University of Tripoli, Tripoli, Libya; 2 Medical Microbiology and Immunology Department, Faculty of Pharmacy, University of Tripoli, Tripoli, Libya; 3 Biochemistry Department, Faculty of Medicine, University of Tripoli, Tripoli, Libya; 4 National Centre for Disease Control, Tripoli, Libya; 5 Faculty of Medical Technology, University of Tripoli, Tripoli, Libya; 6 Laboratoire de Microorganismes et Biomolécules Actives Faculté des Sciences de Tunis, Université de Tunis El-Manar, 2098 El-Manar II, Tunisie.; 7 Laboratoire de Recherche Sciences et Technologies de l’Environnement, Institut Supérieur des Sciences et Technologies de l’Environnement de Borj-Cedria, Université de Carthage, Technopôle de Borj-Cedria, BP-1003, Hammam-Lif 2050, Tunisie.

**Keywords:** AmpC, ESBL, *Klebsiella*, *E. coli*, Libya

## Abstract

**Introduction:**

Extended-spectrum β-lactamases (ESBLs), including the AmpC type, are important mechanisms of resistance among *Klebsiella pneumoniae* and *Escherichia coli* isolates.

**Objective:**

The aim of the study was to investigate the occurrence of AmpC-type β-lactamase producers isolated from two hospitals in Tripoli, Libya.

**Methods:**

All clinical isolates (76 *K. pneumoniae* and 75 *E. coli*) collected over two years (2013-2014) were evaluated for susceptibility to a panel of antimicrobials and were analyzed phenotypically for the ESBL and AmpC phenotype using E-test and ESBL and AmpC screen disc test. Both ESBL and AmpC-positive isolates were then screened for the presence of genes encoding plasmid-mediated AmpC β-lactamases by polymerase chain reaction (PCR).

**Results:**

Of the *K. pneumoniae* and *E. coli* tested, 75% and 16% were resistant to gentamicin, 74% and 1.3% to imipenem, 71% and 12% to cefoxitin, 80% and 12% to cefepime, 69% and 22.6% to ciprofloxacin, respectively. None of the *E. coli* isolates were multidrug resistant compared with *K. pneumoniae* (65.8%). *K. pneumoniae* ESBL producers were significantly higher (85.5%) compared with (17.3%) *E. coli* isolates (P <0.0001, OR=4.93). Plasmid-mediated AmpC genes were detected in 7.9% of *K. pneumoniae*, and 4% *E. coli* isolates. There was low agreement between phenotypic and genotypic methods, phenotypic testing underestimated detection of AmpC enzyme and did not correlate well with molecular results. The gene encoding CMY enzyme was the most prevalent (66.6%) of AmpC positive isolates followed by MOX, DHA and EBC. Only one AmpC gene was detected in 5/9 isolates, i.e, *bla*_CMY_ (n=3), *bla*_MOX_ (n=1), *bla*_DHA_ (n=1). However, co-occurrence of AmpC genes were evident in 3/9 isolates with the following distribution: *bla*_CMY_ and *bla*_EBC_ (n=1), and *bla*_CMY_ and *bla*_MOX_ (n=2). Neither *bla*_FOX_ nor *bla*_ACC_ was detected in all tested isolates. All AmpC positive strains were resistant to cefoxitin and isolated from patients admitted to intensive care units.

**Conclusion:**

Further studies are needed for detection of other AmpC variant enzyme production among such isolates. Continued surveillance and judicious antibiotic usage together with the implementation of efficient infection control measures are absolutely required.

## 1 INTRODUCTION

β-Lactamase production is the predominant mechanism for resistance to β-lactams in *Enterobacteriaceae*. Extended-spectrum β-lactamases (ESBLs) have been reported globally, most often in *Escherichia coli* and *Klebsiella pneumoniaee*^1^. ESBL-producing *K. pneumoniaee* have spread quickly and pose a serious risk of healthcare-associated infections. There is limited data regarding the molecular epidemiology of ESBL-producing *Enterobacteriaceae* in the Middle East and North Africa [1, 2]. AmpC production is one of the mechanisms of resistance to β-lactams in enterobacteria, conferring resistance to all β-lactams except fourth-generation cephalosporins and carbapenems, and is typically associated with multidrug resistance (MDR) [3]. Treatment options are severely limited because AmpC is often associated with other multiple resistance genes, such as those of resistance to quinolones as well as other β-lactamase genes [3, 4]. The genes encoding these enzymes are chromosome or plasmid borne [5]. In particular, *K. pneumoniaee* have acquired plasmid-mediated AmpC β-lactamases [6]. Based on the sequence similarities with species-specific AmpC enzymes, plasmid AmpC variants are classified into five evolutionary groups: the CIT variants (CMY-2 types) originating in *Citrobacter freundii*, the *Enterobacter* sp. EBC variants (ACT-1 type, MIR-1), the *Morganella morganii* DHA variants, the *Hafnia alvei* ACC variants, and the *Aeromonas* sp. FOX and MOX variants [3, 4]. The geographic scattering of the different types of AmpC shows that the CMY-2 type is the most frequent, particularly in Europe [7], and in North Africa [8-10]. In Libya, only a few reports on AmpC production in *Enterobacteriaceae* strains were published [11, 12]. The aim of this study was to investigate the prevalence and molecular epidemiology of cefoxitin resistance *bla*_AmpC_ genes among *K. pneumoniaee* and *E. coli* isolates recovered from hospitalized patients in Tripoli, Libya.

## 2 MATERIALS AND METHODS

### 2.1 Identification and Antibiotic Susceptibility Testing of Isolates

A total of 151 *K. pneumoniaee* and *E. coli* non-duplicate, nonconsecutive isolates were collected during 2013-2014 from two teaching hospitals in Tripoli: Tripoli Medical Centre (TMC) and Tripoli Pediatric Hospital (TPH). All isolates were selected as part of the clinical workup in this prospective laboratory-based surveillance study. Isolated organisms were identified to the species level and tested for their susceptibility to a variety of antimicrobial agents by the BD Phoenix Automated Microbiology System (USA) according to the manufacturer’s instructions.

### 2.2 Phenotypic Detection of ESBL and AmpC

Phenotypic confirmation of ESBLs was carried out using E-test (Liofilchem, Italy). All isolates were initially screened for cefoxitin resistant strains using automated system, then subjected to phenotypic screening for AmpC production using two methods: ESBL and AmpC screen disc kit test (combination disc test [CDT] discs containing cefotaxime alone and in combination with clavulanic acid, cloxacillin and both of these inhibitors are applied) and AmpC E-test (cefotetan/cefotetan+cloxacillin), the AmpC E-test consists of a strip containing cefotetan on one end and cefotetan-cloxacillin on the other end. The results were interpreted and displayed in accordance with manufacturer’s instructions (Liofilchem, Italy) and EUCAST guidelines for detection of resistance mechanisms was implemented, version 5.0 [13]. MDR was defined as showing resistance to three or more different classes of antibiotics such as fluoroquinolones, aminoglycosides, and cephalosporins [14]. Reference strain of *E. coli* ATCC 25922, *E. coli* ATCC 35218 and *K. pneumoniaee* ATCC 700603 were used as controls. In this investigation, specimens were collected under approved ethical standards and the study was reviewed and approved by the Faculty of Pharmacy, University of Tripoli and hospitals participating in this study.

### 2.3 Molecular Detection of *bla_AmpC_* Genes

All isolates were screened for the presence of genes encoding AmpC β-lactamases by polymerase chain reaction (PCR) using previously reported primers [15, 16]. The plasmids were isolated using the QIAGEN Plasmid Mini Kit (Qiagen, Valencia, CA), according to the manufacturer's instructions. The reaction mixture contained a total of 25 µl: 5 µl of 5X Red Load Taq Mix composed of Taq Polymerase, 0.05 µ/µl dNTPs (200 µM) (dATP, dCTP, dGTP, dTTP) reaction buffer with KCl and MgCl2 (1.5 mM) red dye, gel loading buffer, stabilizers (Metabion, Martinsried- Germany); 0.5 µl of each primer 10pmol/µl; primers and extracted plasmid DNA (2-50ng). The thermal profile included one cycle of initial denaturation at 95^o^C for 2 min followed by 35 of denaturation cycles at 95^o^C for 30 sec, annealing at 52^o^C for 30 sec, and extensions at 72^o^C for 45 sec. The PCR reaction was carried out with TC-412 thermocycler (Techne, Duxford, Cambridge). Five µl of the PCR amplification products were electrophoresed in 2% m/v agarose containing 0.5 µg/mL ethidium bromide. The amplified PCR products were visualized under UV light and electronically documented with a gel documentation system (MultiDoc-It Digital Imaging System UVP, Cambridge, UK). A 100bp DNA ladder (Qiagen, Valencia, CA) was used as a molecular size marker.

## 3 RESULTS

Of the *K. pneumoniae* and *E. coli* tested, 75% and 16% were resistant to gentamicin, 74% and 1.3% to imipenem, 71% and 12% to cefoxitin, 80% and 12% to Cefepime, 69% and 22.6% to ciprofloxacin, respectively. The isolates remained susceptible to colistin (Table **1**). None of the *E. coli* isolates were defined as MDR compared with *K. pneumoniae* (50/76; 65.8%). The incidence of ESBL producers was significantly higher among *K. pneumoniae* 65/76 (85.5%) compared with 13/75 (17.3%) of *E. coli* isolates (P <0.0001, OR=4.93). Using PCR, plasmid-mediated AmpC genes were detected in 7.9% (6/76) of *K. pneumoniae*, and 4% (3/75) in *E. coli* isolates Fig. (**1**). Therefore, phenotypic detection of AmpC was only presented for these nine isolates, the origin and characteristics of clinical interest of these isolates are summarized in Table **2**. Only 4/9 (44.4%) were positive using E-test and 3/9 (33.3%) for AmpC screen test disk test. These methods failed to detect one isolate (false-negative) even though the isolates was AmpC positive using PCR. These results demonstrate that phenotypic testing assays on these isolates underestimated detection of AmpC enzyme production and did not correlate well with molecular results. The performance of different AmpC confirmatory tests in combination with different antibiotic and inhibitor combinations is shown in Table **2**.

Table **3** shows the distribution of antibiotic resistance among AmpC positive isolates. All AmpC positive strains were resistant to cefoxitin and the majority were resistant (7/9; 77.8%) to cefepime and recovered from patients hospitalized in different intensive care units (ICUs). The majority of AmpC positive isolates 6/9; 66.6% (4 in *K. pneumoniae*, and 2 in *E. coli*) were found to carry the CMY encoding gene. A single AmpC gene was detected in 5/9 isolates, i.e, *bla*_CMY_ (n=3),*bla*_MOX_ (n=1), *bla*_DHA_ (n=1) however, multiple AmpC genes were detected in 3/9 isolates with the following distribution: *bla*_CMY_ and *bla*_EBC_ (n=1), and *bla*_CMY_ and *bla*_MOX_ (n=2). Neither *bla*_FOX_ nor *bla*_ACC_ was detected in all tested isolates. None of the tested six encoding gene variants were detected in one isolate (Table **4**).

Fig. (**1**). Amplification of plasmid-mediated AmpC gene in ESBL producing K. pneumonia isolates (1-6) by single PCR Lane M 50bp ladder; Lanes 1 to 6 AmpC-producing isolates, lane 7 is positive control. The amplified amplicon size is 481bp.

## 4 DISCUSSION

The occurrence, types and rate of dissemination of AmpC enzymes has increased worldwide, their early detection is crucial and critical since AmpC β-lactamases show marked variation in geographic distribution [17]. Detection of pathogens producing AmpC β-lactamases is often associated with potentially fatal laboratory reports of false susceptibility to β-lactams phenotypically [18]. Thus, their accurate, authentic and valid detection are important from epidemiological, clinical, laboratory, and infection control methods, especially in developing countries. In the present study, we investigated the incidence of plasmid-mediated AmpC among *K. pneumoniae* and *E. coli* clinical isolates from Tripoli hospitals in Libya.

*K. pneumoniae* isolates were found less susceptible to all antimicrobial agents tested and none of *E. coli* isolates were defined as MDR compared with *K. pneumoniae* (65.8%). In contrast to the previous study, we found that 33.2% of *E. coli* and 42% of *K. pneumoniae* were defined as MDR [12]. This study showed that the incidence of ESBL producers was significantly higher among *K. pneumoniae* (85.5%) compared with (17.3%) of *E. coli* isolates. These results indicate that *K. pneumoniae* strains represent a major therapeutic and epidemiological threat and require the implementation of strict hygiene procedures and regular surveillance studies to determine the genetic basis of resistance.

There was low agreement between genotypic and phenotypic methods used in this study, only 4/9; 44.4% and 3/9; 33.3% of genetically identified AmpC producers were found phenotypically positive using E-test for combined disc diffusion method respectively. Therefore, the genotypic and phenotypic methods used for detection of AmpC did not correlate well. Detecting plasmid mediated AmpC with co-existing ESBL, ampC gene was not functional and/or expressed at low levels is very challenging. Given these difficulties in detecting plasmid mediated AmpC β-lactamases, their prevalence is currently being underestimated. Bolmstrom and colleagues showed that the overall sensitivity and specificity were 88 to 93% using E-test strips for detection of AmpC [19, 20]. Similar to our technique using cefepime alone and in combined with β-lactam inhibitor has been evaluated previously and the authors found that this method was the most sensitive test (66.1%) for AmpC co-producers [21]. It has been suggested that the most convenient method for detection of AmpC was the double-disk test [22, 23]. Cefoxitin insusceptibility is a useful screen for *Klebsiella* spp., *Salmonella* spp., *C*. *koseri*, *P*. *mirabilis*, and *E*. *coli* in areas where the ACC-1 and ACC-4 enzymes are not encountered (so far not detected in Libya). Phenotypic detection of AmpC in *E*. *coli* does not indicate if the enzyme is chromosomal or plasmid mediated, but as a crude guide, lack of multiple drug resistance is suggestive of a chromosomal AmpC whereas multiple drug resistance is consistent with either plasmid-mediated or chromosomal AmpC production [24]. Therefore, phenotypic tests cannot distinguish between the various families of plasmid-mediated AmpC enzymes and may also overlook chromosomally determined AmpC β-lactamases with an extended spectrum and hence, it usually poses a problem due to misleading results [25, 26]. Recently, khari and colleagues evaluated different AmpC confirmatory testes showed that there was low agreement between the genotypic and phenotypic detection of AmpC β-lactamases, and suggested that the phenotypic detection of AmpC β-lactamase production has been hampered by the lack of validated methods [27]. Furthermore, phenotypic detection of plasmid-mediated AmpC β-lactamases has been described to have poor specificity and is not advisable for routine detection of these β-lactamases [28]. In contrast to these authors, Reuland and co-authors found not the E-test but double disk combination test cloxacillin as the best test, with the best sensitivity and specificity after the combination of screening criteria, the authors suggested that the difference might be due to differences in the selection of strains [29].

Overall, AmpC gene was detected in 7.9% of *K. pneumoniae*, and 4% in *E. coli* isolates, all isolates were recovered from different ICU patients and mainly from Pediatric hospital. The majority of AmpC positive isolates 66.6% were found to carry CMY encoding gene followed by MOX; DHA and EBC. Our findings are consistent with previous reports noting the predominance of CMY worldwide [30-32]. In accordance with the earlier study in Tunisia reported the coexistence of various *bla*_AmpC_ genes in a single strain and of such coorcurrence in several species in *Enterobacteriaceae* [33], multiple AmpC genes were detected in one-third of isolates: one isolate expressing *bla*_CMY_ and *bla*_EBC_ genes co-exist together, the other two isolates co-expressing *bla*_CMY_ and *bla*_MOX_ genes. However, in 5/9 isolates only one AmpC gene was detected *bla*_CMY_, *bla*_MOX_ or *bla*_DHA_. Similar to our findings neither *bla*_FOX_ nor *bla*_ACC_ were detected in all tested isolates in Algiers hospitals [34]. Two studies in Egypt reported that no genes belonging to ACC were detected in all tested isolates 35-37.None of the targeted six encoding gene variant primers used in the present study were detected in one isolate or indicates they were most likely AmpC hyperproducers that showed positive results phenotypically using one technique because of overexpression of the chromosomal AmpC gene. To our knowledge, this is the first description of these genes in Libyan hospitals.

The shortcoming of the study was multiplex PCR was not performed; detection of plasmid mediated AmpC β-lactamases and other AmpC variants were not investigated. This emphasizes the need for such enzymes detection for preventing this emerging resistance into hospitals and for controlling its spread within the com-munity. That will avoid therapeutic failures and nosocomial outbreaks.

## CONCLUSION

PCR is the gold standard method for detection of AmpC β-lactamase. The dissemination of cefoxitin resistance genes within the hospitals may indicate nosocomial healthcare issue. The most prevalent AmpC gene belongs to CMY followed by MOX; DHA and EBC. Hence, identification of types of AmpC may help the physician to prescribe the most appropriate antibiotic, thus decreasing the selective pressure, which generates antibiotic resistance.

## Figures and Tables

**Fig. (1):**
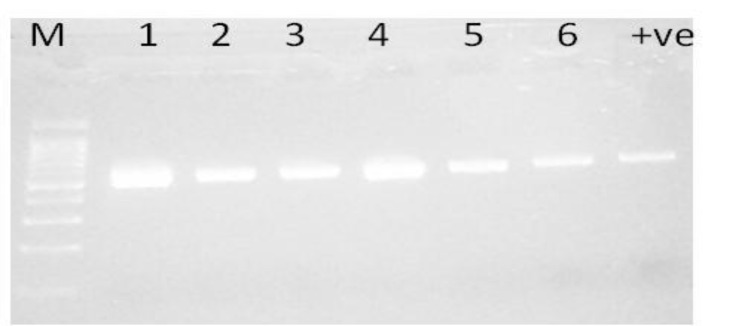
Amplification of plasmid-mediated AmpC gene in ESBL producing *K. pneumoniae* isolates (1-6) by single PCR.

**Table 1:** Antibiotic resistance of *K. pneumoniae* and *E. coli* isolated from different clinical specimens.

**Antibiotic**	***K. pneumoniae*** **n=76 (%)**	***E. coli*** **n= 75 (%)**
Amikacin	14 (18)	0 (0)
Gentamicin	57 (75)	12 (16)
Ertapenem	56 (73)	0 (0)
Imipenem	36 (47)	1 (1.3)
Meropenem	39 (51)	0 (0)
cefoxitin	54 (71)	9 (12)
ceftazidime	63 (82)	10 (13.3)
Ceftriaxone	64 (84)	10 (13.3)
Cefepime	61 (80)	9 (12)
Aztreonam	63 (82)	11 (14.6)
Ampicillin	76 (100)	57 (76)
Amoxicilli-clavulanate	67 (88)	44 (58.6)
Piperacillin-Tazobactam	60 (78)	4 (5.3)
Colistin	0 (0)	0 (0)
Trimetoprim-sulfametoxazol	36 (47)	38 (50)
Nitrofurantoin	55 (72)	1 (1.3)
Ciprofloxacin	53 (69)	17 (22.6)
Levofloxacin	47 (61)	17 (22.6)
MDR	50 (65.8)	0 (0)
ESBL	65 (85.5)	13 (17.3)

**Table 2:** Characteristics of the nine isolates harboring AmpC gene.

**Isolate No.**	**Hospital**	Ward *	**Specimen**±	**age**	**isolate**	**MDR**	**ESBL**	**E-test**	**ESBL/AmpC test^†^**
1	TMC	GSICU	ETT	30 years	*K. pneumoniae*	+	+	-	-
2	TPH	SCBU	Tip	45 days	*K. pneumoniae*	+	+	+	-
3	TPH	ICU	urine	5 years	*K. pneumoniae*	-	+	-	+
4	TPH	SCBU	blood	6 days	*K. pneumoniae*	-	+	+	+
5	TPH	SCBU	umbilical	5 days	*K. pneumoniae*	-	+	+	-
6	TPH	ICU	ETT	14 months	*K. pneumoniae*	-	+	-	-
7	TPH	NBICU	urine	10 years	*E. coli*	-	+	+	-
8	TPH	SCBU	blood	3 years	*E. coli*	-	+	-	+
9	TPH	SCBU	ETT	1 day	*E. coli*	-	+	-	-

* GSICU = general systems intensive care unit; SCBU = special care baby unit; ICU = intensive care unit; NBICU = Newborn intensive care unit ± ETT = Endotracheal tube; † Combination Disc test = discs containing cefotaxime alone and in combination with clavulanic acid, cloxacillin and both of these inhibitors are applied

**Table 3:** Antibiotic resistance of AmpC producers *K. pneumoniae* and *E. coli* isolates.

**Antibiotic**	***K. pneumoniae*** **n=6 (%)**	***E. coli*** **n= 3 (%)**
Amikacin	0 (0)	0 (0)
Gentamicin	4 (66.6)	1 (16)
Ertapenem	2 (33.3)	0 (0)
Imipenem	2 (33.3)	1 (33.3)
Meropenem	2 (33.3)	0 (0)
cefoxitin	6 (100)	3 (100)
ceftazidime	6 (100)	2 (66.6)
Ceftriaxone	6 (100)	3 (13.3)
Cefepime	5 (83.3)	2 (66.6)
Aztreonam	6 (100)	3 (100)
Ampicillin	6 (100)	3 (100)
Amoxicilli-clavulanate	6 (88)	3 (100)
Piperacillin-Tazobactam	3 (50)	1 (33.3)
Colistin	0 (0)	0 (0)
Trimetoprim-sulfametoxazol	2 (33.3)	3 (100)
Nitrofurantoin	3 (50)	0 (0)
Ciprofloxacin	3 (50)	0 (0)
Levofloxacin	3 (50)	0 (0)

**Table 4:** The distribution of the six plasmid-encoded AmpC variant genes.

**Isolate No.**	**isolate**	**Plasmid-mediated AmpC variant genes**						
		***bla*_AmpC_**	***bla*_CMY_**	***bla*_MOX_**	***bla*_DHA_**	***bla*_EBC_**	***bla*_FOX_**	***bla*_ACC_**
1	*K. pneumoniae*	+	+	-	-	+	-	-
2	*K. pneumoniae*	+	-	+	-	-	-	-
3	*K. pneumoniae*	+	-	-	+	-	-	-
4	*K. pneumoniae*	+	+	-	-	-	-	-
5	*K. pneumoniae*	+	+	-	-	-	-	-
6	*K. pneumoniae*	+	+	+	-	-	-	-
7	*E. coli*	+	+	-	-	-	-	-
8	*E. coli*	+	-	-	-	-	-	-
9	*E. coli*	+	+	+	-	-	-	-

## References

[1] Dahmen S., Bettaieb D., Mansour W., Boujaafar N., Bouallègue O., Arlet G. (2010). Characterization and molecular epidemiology of extended-spectrum β-lactamases in clinical isolates of *Enterobacteriaceae* in a Tunisian University Hospital.. Microb. Drug Resist..

[2] Storberg V. (2014). ESBL-producing *Enterobacteriaceae* in Africa - a non-systematic literature review of research published 2008-2012.. Infect. Ecol. Epidemiol..

[3] Philippon A., Arlet G., Jacoby G.A. (2002). Plasmid-determined AmpC-type β-lactamases.. Antimicrob. Agents Chemother..

[4] Jacoby G.A. (2009). AmpC β-lactamases.. Clin. Microbiol. Rev..

[5] Pérez-Pérez F.J., Hanson N.D. (2002). Detection of plasmid-mediated AmpC beta-lactamase genes in clinical isolates by using multiplex PCR.. J. Clin. Microbiol..

[6] Tofteland S., Dahl K.H., Aasnæs B., Sundsfjord A., Naseer U. (2012). A nationwide study of mechanisms conferring reduced susceptibility to extended-spectrum cephalosporins in clinical *Escherichia coli* and *Klebsiella* spp. isolates.. Scand. J. Infect. Dis..

[7] Miró E., Agüero J., Larrosa M.N., Fernández A., Conejo M.C., Bou G., González-López J.J., Lara N., Martínez-Martínez L., Oliver A., Aracil B., Oteo J., Pascual A., Rodríguez-Baño J., Zamorano L., Navarro F. (2013). Prevalence and molecular epidemiology of acquired AmpC β-lactamases and carbapenemases in Enterobacteriaceae isolates from 35 hospitals in Spain.. Eur. J. Clin. Microbiol. Infect. Dis..

[8] Iabadene H., Messai Y., Ammari H., Alouache S., Verdet C., Bakour R., Arlet G. (2009). Prevalence of plasmid-mediated AmpC beta-lactamases among *Enterobacteriaceae* in Algiers hospitals.. Int. J. Antimicrob. Agents.

[9] Wassef M., Behiry I., Younan M., El Guindy N., Mostafa S., Abada E. (2014). Genotypic identification of AmpC β-lactamases production in gram-negative *Bacilli* isolates.. Jundishapur J. Microbiol..

[10] Mnif B., Ktari S., Chaari A., Medhioub F., Rhimi F., Bouaziz M., Hammami A. (2013). Nosocomial dissemination of *Providencia stuartii* isolates carrying *bla* OXA-48, *bla* PER-1, *bla* CMY-4 and *qnrA6* in a Tunisian hospital.. J. Antimicrob. Chemother..

[11] Ahmed S.F., Ali M.M., Mohamed Z.K., Moussa T.A., Klena J.D. (2014). Fecal carriage of extended-spectrum β-lactamases and AmpC-producing *Escherichia coli* in a Libyan community.. Ann. Clin. Microbiol. Antimicrob..

[12] Abujnah AA, Zorgani A, Sabri MA (2015). Multidrug resistance and extended-spectrum β-lactamases genes among *Escherichia coli* from patients with urinary tract infections in Northwestern Libya.. Libyan J. Med..

[13] The European Committee on Antimicrobial Susceptibility Testing. EUCAST. Breakpoint tables for interpretation of MICs and zone diameters. Version 5.0. 2015; Available at: http://www.eucast.org/fileadmin/src/media/PDFs/EUCAST_files/Breakpoint_tables/v_5.0_ Breakpoint_Table_01.pdf. http://www.eucast.org/fileadmin/src/media/PDFs/EUCAST_files/Breakpoint_tables/v_5.0_Breakpoint_Table_01.pdf.

[14] Magiorakos A.P., Srinivasan A., Carey R.B., Carmeli Y., Falagas M.E., Giske C.G., Harbarth S., Hindler J.F., Kahlmeter G., Olsson-Liljequist B., Paterson D.L., Rice L.B., Stelling J., Struelens M.J., Vatopoulos A., Weber J.T., Monnet D.L. (2012). Multidrug-resistant, extensively drug-resistant and pandrug-resistant bacteria: an international expert proposal for interim standard definitions for acquired resistance.. Clin. Microbiol. Infect..

[15] Soltan Dallal M.M., Sabbaghi A., Molla Agha H. (2013). Prevalence of AmpC and SHV β-lactamases in clinical isolates of *Escherichia coli* from Tehran hospitals.. Jundishapur J. Microbiol..

[16] Geyer C.N., Hanson N.D. (2014). Multiplex high-resolution melting analysis as a diagnostic tool for detection of plasmid-mediated AmpC β-lactamase genes.. J. Clin. Microbiol..

[17] Gupta G., Tak V., Mathur P. (2014). Detection of AmpC β-lactamases in gram-negative bacteria.. J. Lab. Physicians.

[18] Ding H., Yang Y., Lu Q., Wang Y., Chen Y., Deng L., Wang A., Deng Q., Zhang H., Wang C., Liu L., Xu X., Wang L., Shen X. (2008). The prevalence of plasmid-mediated AmpC β-lactamases among clinical isolates of *Escherichia coli* and *Klebsiella pneumoniaee* from five children’s hospitals in China.. Eur. J. Clin. Microbiol. Infect. Dis..

[19] Bolmstrom A., Engelhardt A., Bylund L., Ho P., Karlsson A. (2006). Evaluation of two new etest strips for AmpC detection, abstr. D-0451. Abstr.. 46th Interscinces Conference on Antimicrobial Agents and Chemotherapy. 2006; Available at: http://www.eucast.org/fileadmin/src/media/PDFs/ EUCAST_files/Breakpoint_tables/v_5.0_Breakpoint_Table_01.pdf.

[20] Engelhardt A., Yusof A., Ho P. (2008). Evaluation of a new Etest strip for AmpC detection using a large collection of genotypically characterized strains, abstr. D-280. Abstr.. 48th Interscinces Conference on Antimicrobial Agents and Chemotherapy.

[21] Kaur J., Mahajan G., Chand K., Sheevani, Chopra S. (2016). Enhancing phenotypic detection of ESBL in AmpC co-producers by using cefepime and tazobactam.. J. Clin. Diagn. Res..

[22] Moland E.S., Kim S.Y., Hong S.G., Thomson K.S. (2008). Newer beta-lactamases: clinical and laboratory implications, part II.. Clin. Microbiol. Newsl..

[23] Ruppé E., Bidet P., Verdet C., Arlet G., Bingen E. (2006). First detection of the Ambler class C 1 AmpC beta-lactamase in *Citrobacter freundii* by a new, simple double-disk synergy test.. J. Clin. Microbiol..

[24] Thomson K.S. (2010). Extended-spectrum-β-lactamase, AmpC, and Carbapenemase issues.. J. Clin. Microbiol..

[25] Mammeri H., Eb F., Berkani A., Nordmann P. (2008). Molecular characterization of AmpC-producing *Escherichia coli* clinical isolates recovered in a French hospital.. J. Antimicrob. Chemother..

[26] Handa D., Pandey A., Asthana A.K., Rawat A., Handa S., Thakuria B. (2013). Evaluation of phenotypic tests for the detection of AmpC beta-lactamase in clinical isolates of *Escherichia coli.*. Indian J. Pathol. Microbiol..

[27] Mohd Khari F.I., Karunakaran R., Rosli R., Tee Tay S. (2016). Genotypic and phenotypic detection of AmpC β-lactamases in *Enterobacter spp.* Isolated from a Teaching Hospital in Malaysia.. PLoS One.

[28] Reuland E.A., Halaby T., Hays J.P., de Jongh D.M., Snetselaar H.D., van Keulen M., Elders P.J., Savelkoul P.H., Vandenbroucke-Grauls C.M., Al Naiemi N. (2015). Plasmid-mediated AmpC: prevalence in community-acquired isolates in Amsterdam, the Netherlands, and risk factors for carriage.. PLoS One.

[29] Reuland E.A., Hays J.P., de Jongh D.M., Abdelrehim E., Willemsen I., Kluytmans J.A., Savelkoul P.H., Vandenbroucke-Grauls C.M., al Naiemi N. (2014). Detection and occurrence of plasmid-mediated AmpC in highly resistant gram-negative rods.. PLoS One.

[30] Yilmaz N.O., Agus N., Bozcal E., Oner O., Uzel A. (2013). Detection of plasmid-mediated AmpC β-lactamase in *Escherichia coli* and *Klebsiella pneumoniaee*.. Indian J. Med. Microbiol..

[31] Park M.J., Kim T.K., Song W., Kim J.S., Kim H.S., Lee J. (2013). An Increase in the clinical isolation of acquired AmpC β-lactamase-producing *Klebsiella pneumoniaee* in Korea from 2007 to 2010.. Ann. Lab. Med..

[32] Denisuik A.J., Lagacé-Wiens P.R., Pitout J.D., Mulvey M.R., Simner P.J., Tailor F., Karlowsky J.A., Hoban D.J., Adam H.J., Zhanel G.G., Canadian Antimicrobial Resistance Alliance (2013). Molecular epidemiology of extended-spectrum β-lactamase-, AmpC β-lactamase- and carbapenemase-producing *Escherichia coli* and *Klebsiella pneumoniaee* isolated from Canadian hospitals over a 5 year period: CANWARD 2007-11.. J. Antimicrob. Chemother..

[33] Gharout-Sait A., Touati A., Guillard T., Brasme L., de Champs C. (2015). Molecular characterization and epidemiology of cefoxitin resistance among *Enterobacteriaceae* lacking inducible chromosomal ampC genes from hospitalized and non-hospitalized patients in Algeria: description of new sequence type in *Klebsiella pneumoniaee* isolates.. Braz. J. Infect. Dis..

[34] Chérif T., Saidani M., Decré D., Boutiba-Ben Boubaker I., Arlet G. (2015). Cooccurrence of Multiple AmpC β-Lactamases in *Escherichia coli, Klebsiella pneumoniaee*, and *Proteus mirabilis* in Tunisia.. Antimicrob. Agents Chemother..

[35] Iabadene H., Messai Y., Ammari H., Alouache S., Verdet C., Bakour R., Arlet G. (2009). Prevalence of plasmid-mediated AmpC β-lactamases among *Enterobacteriaceae* in Algiers hospitals.. Int. J. Antimicrob. Agents.

[36] El-Hady S., Adel L. (2015). Occurrence and detection of AmpC β-lactamases among *Enterobacteriaceae* isolates from patients at Ain Shams University Hospital.. Egypt. J. Med. Hum. Genet..

[37] Helmy M.M., Wasfi R. (2014). Phenotypic and molecular characterization of plasmid mediated AmpC β-lactamases among *Escherichia coli, Klebsiella* spp., and *Proteus mirabilis* isolated from urinary tract infections in Egyptian hospitals.. BioMed Res. Int..

